# Identification and Characterization of *Erysiphe necator*-Responsive MicroRNAs in Chinese Wild *Vitis pseudoreticulata* by High-Throughput Sequencing

**DOI:** 10.3389/fpls.2016.00621

**Published:** 2016-05-31

**Authors:** Lijuan Han, Kai Weng, Hui Ma, Gaoqing Xiang, Zhiqian Li, Yuejin Wang, Guotian Liu, Yan Xu

**Affiliations:** ^1^State Key Laboratory of Crop Stress Biology for Arid Areas, College of Horticulture, Northwest A&F UniversityYangling, China; ^2^College of Horticulture, Northwest A&F UniversityYangling, China; ^3^Key Laboratory of Horticultural Plant Biology and Germplasm Innovation in Northwest China, Ministry of Agriculture, College of Horticulture, Northwest A& F UniversityYangling, China

**Keywords:** Chinese wild *Vitis pseudoreticulata*, high-throughput sequencing, miRNA identification, powdery mildew resistance, target genes

## Abstract

Grapevine powdery mildew is one of the most damaging fungal diseases. Therefore, a precise understanding of the grapevine disease resistance system becomes a subject of significant importance. Plant microRNAs(miRNAs) have been implicated to play regulatory roles in plant biotic stress responses. In this study, high-throughput sequencing and miRDeep-P were employed to identify miRNAs in Chinese wild *Vitis pseudoreticulata* leaves following inoculation with *Erysiphe necator*. Altogether, 126 previously identified microRNAs and 124 novel candidates of miRNA genes were detected. Among them, 43 conserved miRNAs belong to 20 families and 23 non-conserved but previously-known miRNAs belong to 15 families. Following *E. necator* inoculation, 119 miRNAs were down-regulated and 131 were up-regulated. Furthermore, the expression changes occurring in 32 miRNAs were significant. The expression patterns of some miRNAs were validated by semi-quantitative RT-PCR and qRT-PCR. A total of 485 target genes were predicted and categorized by Gene Ontology (GO). In addition, 14 vvi-miRNAs were screened with 36 targets which may be involved in powdery mildew resistance in grape. Highly accumulated vvi-NewmiR2118 was detected from accession “Baihe-35-1,” whose targets were mostly NBS-LRR resistance genes. It was down-regulated rapidly and strongly in “Baihe-35-1” leaves after inoculated with *E. necator*, indicating its involvement in grape powdery mildew resistance. Finally, the study verified interaction between vvi-NewmiR2118 and *RPP13* by histochemical staining and GUS fluorescence quantitative assay.

## Introduction

Grapevine is one of the world's oldest and most important fruit crops—culturally, nutritionally and economically. However, many important grapevine varieties are susceptible to pathogen infection, such as *Plasmopara viticola* (Richter et al., 2006) and *Erysiphe necator* (Akkurt et al., 2007). The latter is especially detrimental as it occurs in almost all countries and regions where grapes are grown and causes major loss of grape production each year.

Plant miRNAs are a class of ~22-neucleotide(nt) small non-coding RNAs (Reinhart et al., 2002). Since their discovery in *Arabidopsis* (Reinhart et al., 2002), a number of miRNAs have been recorded. Though most miRNAs are involved in plant growth and development (Chen, 2005), recent studies have found that miRNAs also play pivotal roles in plant immunity (Mallory and Vaucheret, 2006; Ruiz-Ferrer and Voinnet, 2009; Sunkar et al., 2012). In plants, the first miRNA reported to show disease resistance activity is ath-miRNA393. When induced by flg22, ath-miR393 down regulated the F-box auxin receptors TIR1, AFB2 and AFB3, which further repressed auxin signaling. As a result, the growth of *P. syringae* was restricted on host plants, implying that miRNA-mediated suppression of auxin signaling enables enhanced disease resistance in plants (Navarro et al., 2006). microRNA398 was down-regulated in response to oxidative stress caused by abiotic or biotic stresses (Sunkar et al., 2006; Jagadeeswaran et al., 2009a). After infiltrated with avirulent strain *avrRpm1* or *avrRpt2*, the expression level of ath-miR398 decreased significantly, but one of its targets Cu/Zn superoxide dismutase cytosolic CSD1 was elevated, indicating that miR398 involves in the biotic stress regulatory networks (Jagadeeswaran et al., 2009a). Another research found that miR160, miR67 and miR393 were up-regulated obviously while miR825 was down-regulated greatly after inoculating *Arabidopsis* with *Pst DC3000 hrcC* for 1 and 3 h (Fahlgren et al., 2007). In both single mutants dcl1 and hen1 whose miRNA synthesis was hindered, spores of *Pst DC3000 hrcC, Pseudomonas syringae pv. Phaseolicola, P. fluorescens* and *Escherichia coli* proliferated faster than on wild type (Navarro et al., 2008). In *Oryza sativa*, novel osa-miR7695 targeted an alternatively spliced transcript OsNramp6, a gene contributing to pathogen resistance (Baldrich et al., 2013; Campo et al., 2013). The researchers of this study believed that alternative splicing might be a mechanism adopted by plants of attenuating miRNA-mediated gene regulation. This speculation is proposed by other researches (Yan et al., 2012; Yang et al., 2012) as well, complicating plant immunity mechanisms based on the miRNA regulation. Not only do natural miRNAs act in plant biotic stress regulatory networks, but also some appropriately modified artificial miRNAs can confer resistance to pathogen susceptible plants, which is elegantly proved by Niu et al. (2006). When researchers modified the ath-miRNA159 precursor into amiR-P69159 and amiR-HC-Pro159 which respectively targets virus proteins P69 and HC-Pro, transgenic *A. thaliana* plants obtained specific resistance to TYMV and TuMV. Moreover, transgenic plants expressing both artificial miRNAs from a modified dimeric miR159 precursor conferred resistance to both viruses. These results implicate the possibility to use amiRNA-mediated approaches to engineer resistance to multiple viruses in crop plants (Niu et al., 2006).

China is one of the principal centers of origin of *Vitis* species. Chinese *Vitis* species, especially wild grapevines have extremely high resistance to many fungi and bacteria diseases. In addition, Chinese *Vitis* species can be easily crossed with *V. vinifera*, conferring the disease resistance from Chinese *Vitis* species into other *V. vinifera* like European grapevines (Wang et al., 1998; He, 1999). For example, *V. pseudoreticulata* accession Baihe-35-1 has been reported several times to be highly resistant to powdery mildew (Wan et al., 2007). Although, some work has been done to analyze the roles of miRNAs in grapevine-pathogen interaction (Alabi et al., 2012), very little is known about miRNA-involved molecular processes that regulate resistance to powdery mildew. In this study, a powdery mildew-resistant accession of Chinese wild *Vitis pseudoreticulata* Baihe-35-1 was used as materials. This study was aimed to profile the expression of powdery mildew responsive miRNAs in Chinese wild grape. Two sRNA libraries were constructed for inoculated and non-inoculated grapevine leaves. Then the illumina platform was employed for miRNA high-throughput sequencing (Solexa sequencing). Computational analysis was done to screen miRNAs relative to powdery mildew resistance. We also used stem-loop real time qRT-PCR to validate miRNAs differentially expressed. This study identified highly accumulated vvi-NewmiR2118 from *V. pseudoreticulata*. Using susceptible cultivars of Piont Noir as control material, we showed that vvi-NewmiR2118 was down-regulated greatly in “Baihe-35-1” leaves after inoculated with *E. necator*, indicating its involvement in grape powdery mildew resistance. Vvi-NewmiR2118 mainly targeted NBS-LRR resistance genes. Then histochemical staining and GUS fluorescence quantitative assay verified that it interacted *RPP13.* This study not only enables a better understanding of host–pathogen interactions but also sheds light on new strategies for breeding disease resistance grapevine varieties.

## Materials and methods

### Plant materials and stress treatments

The Chinese wild *V. pseudoreticulata* accession “Baihe-35-1” used in this study were grown in the Grape Germplasm Resources orchard of Northwest Agriculture and Forest University, Yangling, China. The second to fourth young and healthy leaves from shoot apexes were inoculated with *E. necator* in field conditions. The inoculation was carried out as described by Weng et al. (2014). Briefly, “Baihe35-1” leaves were pressed against grape leaves already infected with powdery mildew and covered immediately with a paper bag. Sample leaves were collected 0, 12, 24, 48, 72, 96, 120, and 144 h after inoculation and stored at −80°C. Three-year old clonal Chinese wild *V. pseudoreticulata* accession “Baihe-35-1” and the susceptible control material Pinot Noir were grown in the climate chamber at 16 h light/8 h dark at 25–26°C for semi-quantitative RT-PCR and real-time qPCR.

### Total RNA extraction and small RNA sequencing

Total RNA was isolated from each frozen sample using the modified SDS-phenol method (Zhang et al., 2003; Wang et al., 2004). The extracted RNA was then treated with DNAseI to remove genomic DNA. The RNA concentration was detected before and after DNAseI treatment using a NanoDropTM 1000 spectrophotometer (Thermo Fisher Scientific, USA), and its integrity was ascertained visually with 0.6% agarose gel electrophoresis.

Mix equal total RNAs from each inoculation time as the treated group. Then 1 mg samples of both treated and control RNAs were subjected to small RNA high-throughput sequencing (HTS) with Solexa sequencing technology at the Beijing Genomics Institute (BGI), Shenzhen, China.

### Predict miRNAs and their targets from *V. pseudoreticulata*

miRDeep-P (Yang and Li, 2011) is an effective tool for plant miRNA searching, so it was used in this study to determine and analyze miRNAs in “Baihe-35-1” leaves. The miRNA prediction was processed as described by Yang and Li (2011). Grapevine Pinot Noir genome (http://bowtie-bio.sourceforge.net/) was used as reference sequences.

Putative mature miRNA sequences were used as queries to search against the grape gene (http://www.genoscope.cns.fr/) using online server psRNATarget (Dai and Zhao, 2011). Alignments between each miRNA and its target(s) should satisfy the following criteria: the maximum expectation is 3.0; the length for complementarity scoring is 20 bp; target accessibility-maximum energy to unpair the target site (UPE) is no more than 25.0; upstream and downstream flanking length around the target site were 17 and 13 respectively; range of central mismatch leading to translational inhibition was 9–11 nucleotides. Further, GO analysis was carried out for target genes by AgriGO (http://bioinfo.cau.edu.cn/agriGO/) (Zhou et al., 2010).

### Expression pattern analysis of miRNAs

In this study, semi-quantitative RT-PCR and stem-loop real-time PCR were employed to display the expression patterns of vvi-miRNAs with 5.8SrRNA as reference gene. The semi-quantitative RT-PCR shared the same primers with stem-loop real-time PCR. The primers were designed following the instructions of Chen (Chen et al., 2005). The 5′ end of reverse transcription primers (RT primers) was universal, while the 3′ end was specific by reverse complement with 7–10 bases from the 3′ end of the mature miRNA. We used Oligo(dt) to reverse transcribe 5.8SrRNA. The forward quantitative primers were similar to mature miRNA sequences with about 9 bases excluded at their 3′ ends and 4–6 bases added at the 5′ ends according to the G/C content. Meanwhile, the universal reverse primer was a part of stem-loop RT primers. All primers were synthesized by the Invitrogen company (Table S1). Total RNAs for expression pattern analysis were extracted from grapevine leaves inoculated with *E. necator* for 0, 12, 24, 48, 72, 96, 120, and 144 h as previously described.

The reverse transcription of the vvi-miRNA was done as follows: prepare reaction mixture of 1.0 μg total RNA, 1.0 μl RT primer (10 μM), and 1.0 μl Oligo(dT) (10 μM) in a RNAse-free PCR tube with RNAse-free water added to a total volume of 10.0 μl. The mixture was then incubated at 80°C for 5 min followed by cooling on ice for 10 min. Then, 10.0 μl mixture (4 μl 5 × M-MLV buffer (*Takara*), 2.5 μl dNTPs (10 mM each, *Takara*), 0.5 μl RNAse inhibitor (40 U/μl) and 1 μl Rtase M-MLV (200 U/μl, *Takara*), RNAse-free water 2.0 μl) was added to the same PCR tube. The 20.0 μl reactions were incubated at 16°C for 30 min, followed by 60 cycles of 20°C for 30 s, 42°C for 30 s and 50°C for 1 s and finally inactivated the reaction at 85°C for 5 min.

Semi-quantitative RT-PCR reactions for vvi-miRNAs were performed for 4 min at 95°C, followed by 30–40 cycles (adjusted for individual vvi-miRNA) of 94°C for 30 s, 56°C for 30 s, 72°C for 30 s and final elongation at 72°C for 5 min. The reaction mixture was 2 × rTaq 10 μl, cDNA 1.0 μl, forward primer (10 μM) 1.0 μl, reverse primer (10 μM) 1.0 μl, ddH_2_0 up to 20μl.

Real-time quantitative PCR was carried out using a SYBR premix Ex TaqII kit protocol on an iQ5_Real-Time System (Bio-Rad). The 21 μl PCR included 2.0 μl RT product, 10 μl 2xSYBR Taq, 0.8 μl forward primer (10 μM), 0.8 μl reverse primer (10 μM) and 7.4 μ l RNAse-free H_2_O. The reactions were incubated in a 96-well plate for 3 min at 95°C, followed by 45 cycles of 95°C for 5 s and 58°C for 30 s. Relative expression was calculated using the 2^−ΔΔCt^ method, normalized to the expression of 5.8S rRNA. Three biological and technical replicates were used for each sample.

### Expression analysis of miRNA targets

The expression of some target genes was analyzed by real-time quantitative PCR. Primers are listed in Table S1. Total RNAs were extracted from “Baihe-35-1” leaves with the same inoculation treatment as previously described. First-strand cDNA was synthesized from 1.0 μg DNase-treated total RNA according to the instruction of PrimeScript II 1st strand kit (Takara). qRT-PCR was carried out on an IQ5 real-time PCR machine (Bio-Rad, Hercules, CA) using SYBR green (Takara Biotechnology), according to the protocol provided. The relative expression was calculated using the 2^−ΔΔCt^ method normalized to the expression of 5.8S rRNA.

### Construction of transient expression vectors

Pro_35S_:*MIR2118* and Pro_35S_:Δ*RPP13-GUS* vectors were constructed to verify interaction between vvi-NewmiR2118 and its target RPP13 (Recognition of Peronospora Parasitica, RPP). *MIR2118* was PCR amplified from “Baihe-35-1” genomic DNA with the primer combinations 5′-ggg GAGCTCCTAGGGTTTTTGCAGAGTAGTATAAAA GG-3′ and 5′-ggg GGTACCGTAG CTTCAAGTTCAAGTCAAATCG AAC-3′. Then *MIR2118* was cloned into vector pCAMBIA1307 harboring the 35S promoter. For the Pro_35S_:Δ*RPP13–GUS* construct, 998bp coding sequence from the start codon of “Baihe-35-1” *RPP13* cDNA was amplified using the primer pair 5′-GGATCCATG GCTTCTTGG-3′ and 5′-GAATTCGTCGGATGT GCACAGGTGTCT-3′, termed ΔRPP13. This includes the interaction regions of vvi-NewmiR2118 and RPP13. ΔRPP13 was then inserted into vector pCAMBIA0390, previously modified by our team by inserting coding sequences of *GUS* after its 35S promoter. All constructs were confirmed with standard sequencing before the correct construct was transformed into Agrobacterium strain GV3101 by electroporation using Eppendorf 4308.

### *Nicotiana benthamiana* transient transformation

For transient analysis of the To verify the interaction of vvi-NewmiR2118 and ΔRPP13, we transiently expressed them through expression vectors constructed above in *Nicotiana benthamiana* by *Agrobacterium tumefaciens* transformation. Plants used grew in a growth chamber at 22°C under 16 h light (Sparkes et al., 2006) for 6 weeks. The healthy third and fourth leaves from the apical meristem were infiltrated by 1 ml needleless syringe filled with *Agrobacterium tumefaciens* (OD_600_ = 0.6) or the control suspension throughout the whole abaxial surface. The transformation were settled as: (A) penetration buffer, (B) *Pro*_35*S*_*:MIR2118*, (C) *Pro*_35*S*_*:GUS*, (D) *Pro*_35*S*_*:*Δ*RPP13-GUS*, (E) *Pro*_35*S*_*:MIR2118* and Pro_35*S*_*:GUS* (v/v = 5:1), and (F) *Pro*_35*S*_*:MIR2118* and *Pro*_35*S*_*:*Δ*RPP13-GUS* (v/v = 5:1) co-transformation (**Figure 10**). The infiltrated leaves were marked before the whole plants were replaced into the growth chamber and cultivated under previous growing conditions before they were collected after 48 h infiltration for GUS staining and fluorescence quantitative assay performed as described by Bradford (1976) and Jefferson et al. (1987). Three biological repeats were performed.

## Results

### Small rans from “Baihe-35-1”

**Figure 1:**
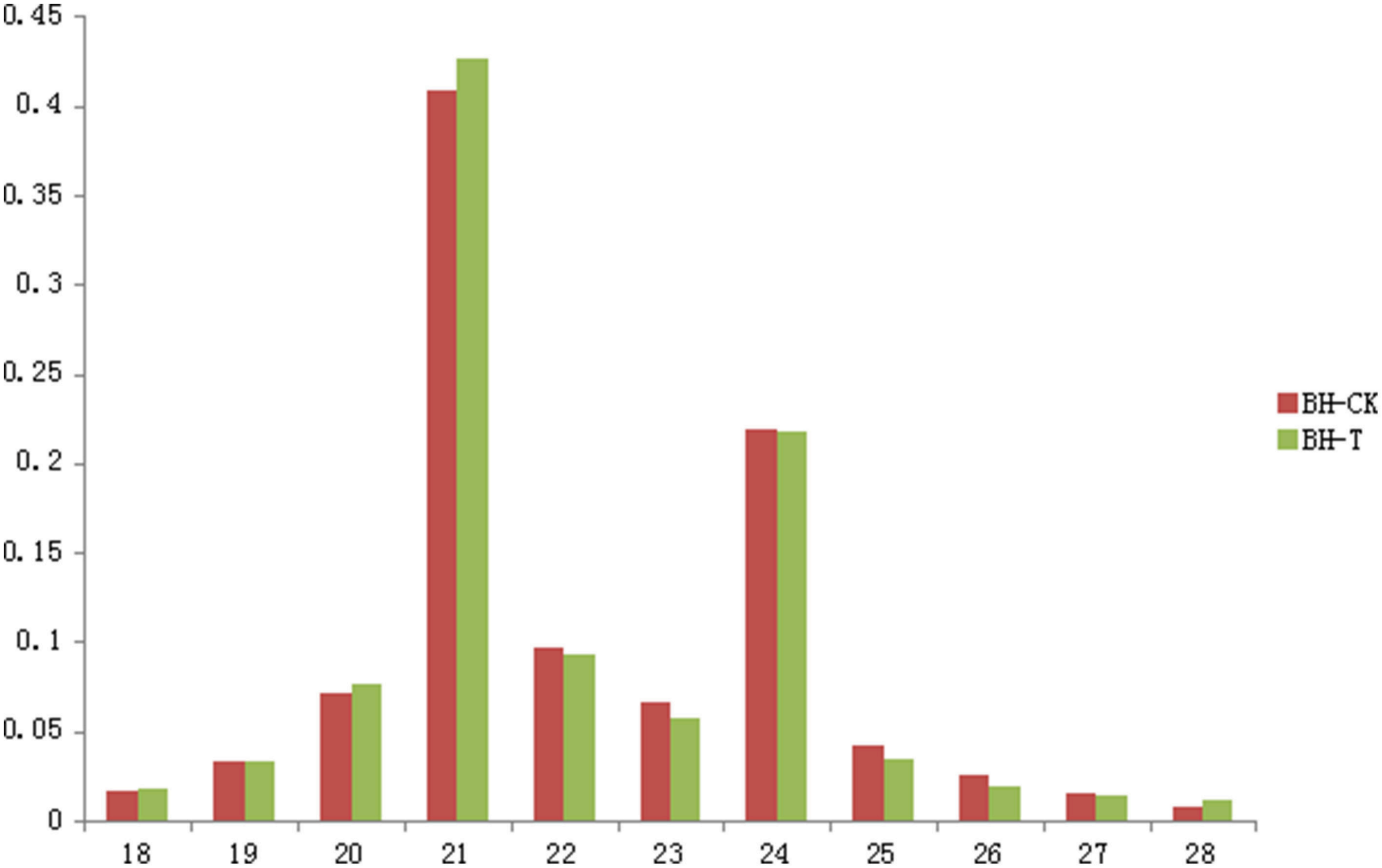
**The length distribution of small RNA sequences in leaves inoculated with ***Erysiphe necator*** and the control**. The x-axis shows nucleotide numbers of small RNA sequences while the y-axis shows the frequency of reads.

To identify miRNAs from “Baihe-35-1,” total RNAs from young leaves inoculated with *E. necator* (see Materials and Methods) and the control were extracted separately for high throughput sequencing, yielding 24839978 and 25714911 raw read totals from the control (BH-C) and treated (BH-T) libraries, respectively (Table 1). After filtration, 24829158 and 25703809 clean reads were obtained, which merged into 3964135 and 3777370 unique reads from BH-C and BH-T libraries. Finally 1626820 reads (41.04% of unique reads) in the BH-C and 1584520 reads (41.95% of unique reads) in the BH-T were mapped onto the *vitis* reference genome, which occupied 6.55% and 6.16% of clean reads, respectively. Length distribution analyses showed that the small RNA length from *V. pseudoreticulata* varied from 18-nt to 27-nt in both the control and the treated group, with two main peaks at 21-nt and 24-nt (Figure 1).

**Table 1:** **Results of small RNA high-throughput sequencing for leaves of ***Vitis pseudoreticulata*** cv “Baihe-35-1**.”

	**BH-C**		**BH-T**	
Total reads	24839978	100.00%	25714911	100.00%
Clean reads	24829158	99.96%	25703809	99.96%
Unique reads	3964135	15.96%	3777370	14.69%
Mapped reads	1626820	6.55%	1584520	6.16%

Total reads, clean reads, unique reads, and mapped reads are referenced to the number of all reads, reads removed of low quality reads, reads getting rid of redundancy and reads mapped on grape genome respectively in each group. BH-CK referred to the control group and BH-T the experimental group.

### vvi-miRNA identification from “Baihe-35-1”

Clean reads from high-throughput sequencing were processed according to miRDeep-P workflow with the *V. vinifera* genome as the reference sequence. There were 250 miRNAs, with 126 known miRNAs and 124 candidates (named vvi-New) (Table S2) identified from Chinese wild *V. pseudoreticulata.* The known miRNAs were classified into 37 families. In our research, 38 known miRNAs (labeled as vvi-NewmiRNA; Table S2) were identical to, or had limited nucleotide shifts from previously reported miRNAs in mature sequences, but were found different stem-loop precursors. Moreover, most known miRNAs from Chinese wild grape were detected in both libraries with 17 detected only in the control, and 15 specific in the treated group (Table S3). Oppositely, most candidates could only be detected from one library (Table S3). However, the read numbers of the candidates were much lower than those of the known miRNAs. Members in different families varied greatly. The largest families were vvi-miR395 and vvi-miR535 with 13 members, followed by vvi-miR156 with 8 members and vvi-miR169 and vvi-miR171 with 7 members each, while seven families (vvi-miR159, vvi-miR162, vvi-miR164, vvi-miR390, vvi-miR397, vvi-miR399, vvi-miR408) only had one member detected. Based on our sequencing, there were significant differences in the expression levels of known miRNAs. The read numbers of most miRNAs were several hundreds. But the expressions of vvi-miR156f,g, vvi-miR166f,g, vvi-miR167b,e,d, vvi-miR3636, and vvi-miR535b,c were extremely high. While several miRNA families or members such as miR156a, miR169d,j, and miR395 had low expression with less than 100 reads detected.

The length of miRNA mature sequences from “Baihe-35-1” were distributed among 18–24 nucleotides. Though most vvi-miRNAs were 21 or 22-nt, the longest was vvi-miR393b with 24 nucleotides and the shortest was 18-nt vvi-miR396a. Family members could be sequenced with different lengths; for example, members of the family miR156 had lengths of 20 and 21-nt, miR167 had lengths of 21 and 22-nt, miR169 and 398 had lengths of 21 and 23-nt.

**Figure 2:**
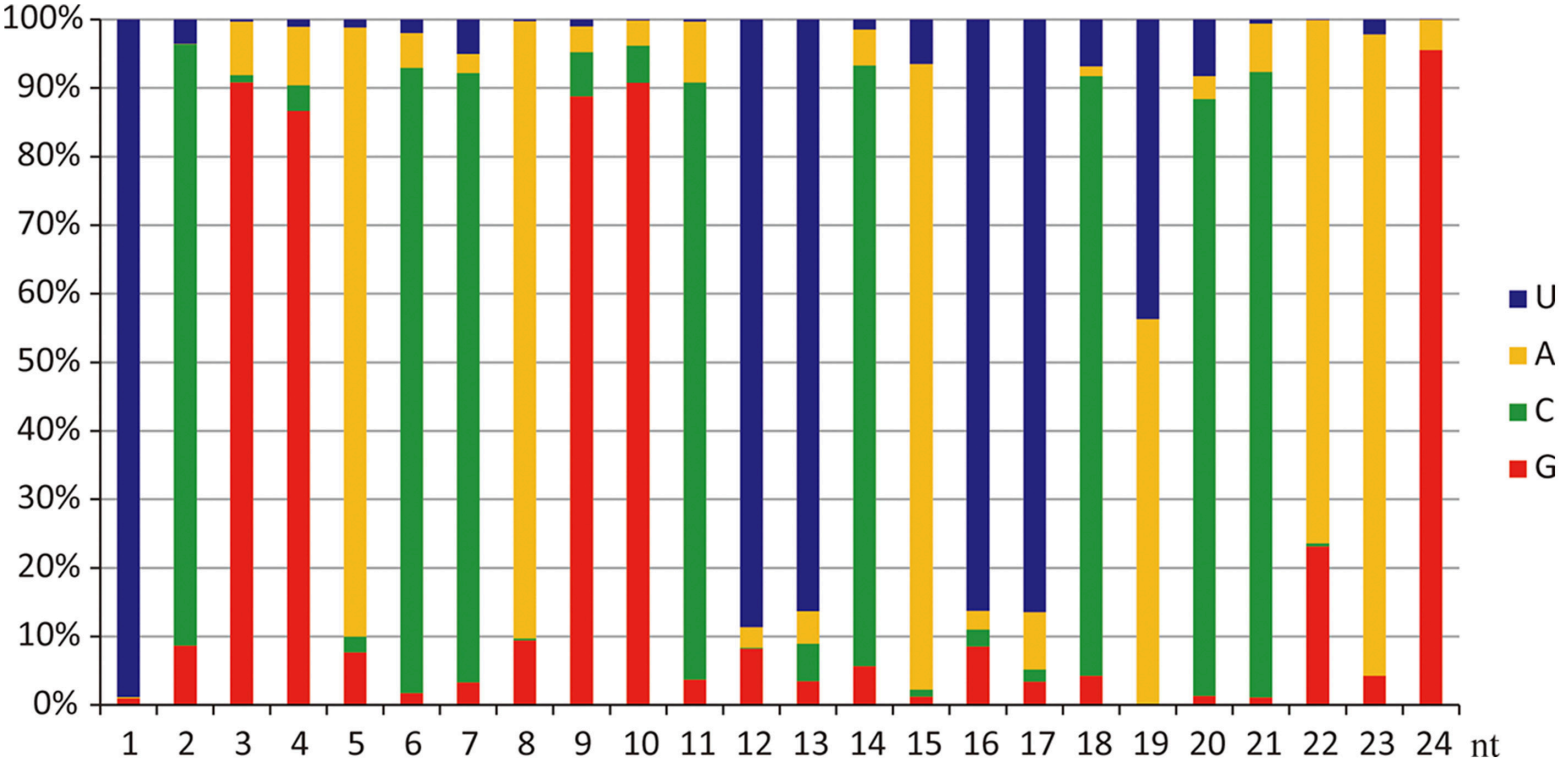
**vvi-miRNA nucleotide bias at each position**. U was dominant at the first position of 5′ end, while C was the most frequently used base in miRNAs of Chinese wild *Vitis pseudoreticulata*. Each site had its favorite ribonucleotide among A, U, C, and G.

Figure 2 shows the nucleotide bias at each position in vvi-miRNAs. In agreement with previous reports, U was dominant at the first position of vvi-miRNAs from Chinese wild *V. pseudoreticulata*, which is a well-established characteristic of miRNAs (Carra et al., 2009), but C was the most frequently used base (about 38.6%). At almost every site of mature vvi-miRNA sequences, there was bias for a specific ribonucleotide among A, U, C, and G. The phenomenon that most A and U were distributed in the front 5′ end of a sequence with G and C more frequent in the 6–18 positions may have some relationship with miRNA function mechanisms, such as binding with corresponding targets (Xu et al., 2010).

### Conserved and non-conserved miRNAs in “Baihe-35-1”

One of the notable characteristics of plant miRNA is its highly-conserved nature. This may be partially explained by its involvement in biological development and stress responses. Unsurprisingly, almost all of the 24 conserved miRNA families (Jones-Rhoades et al., 2006; Rajagopalan et al., 2006) but vvi-miR161, vvi-miR168, vvi-miR391 and vvi-miR394 were detected in our libraries (Table S4). This indicates that the quality of our sRNA high-throughput sequencing is good, and the tools chosen for miRNA identification are also suitable.

In addition to conserved miRNAs, 15 known non-conserved miRNA families were also identified in wild Chinese grape “Baihe-35-1”. Alabi et al. (2012) found seven miRNA families vvi-miR472, vvi-miR529, vvi-miR530, vvi-miR827, vvi-miR894, vvi-miR1507, and vvi-miR1511 that had not been reported previously from grapevine. However, only vvi-miR529, vi-miR530 and vvi-miR827 were detected by us in wild Chinese *V. pseudoreticulata*. But we found that vvi-NewmiR2118 was highly expressed with 37338 reads in the control and 25426 reads in the inoculated samples.

### Target prediction of vvi-miRNAs from “Baihe-35-1”

**Figure 3:**
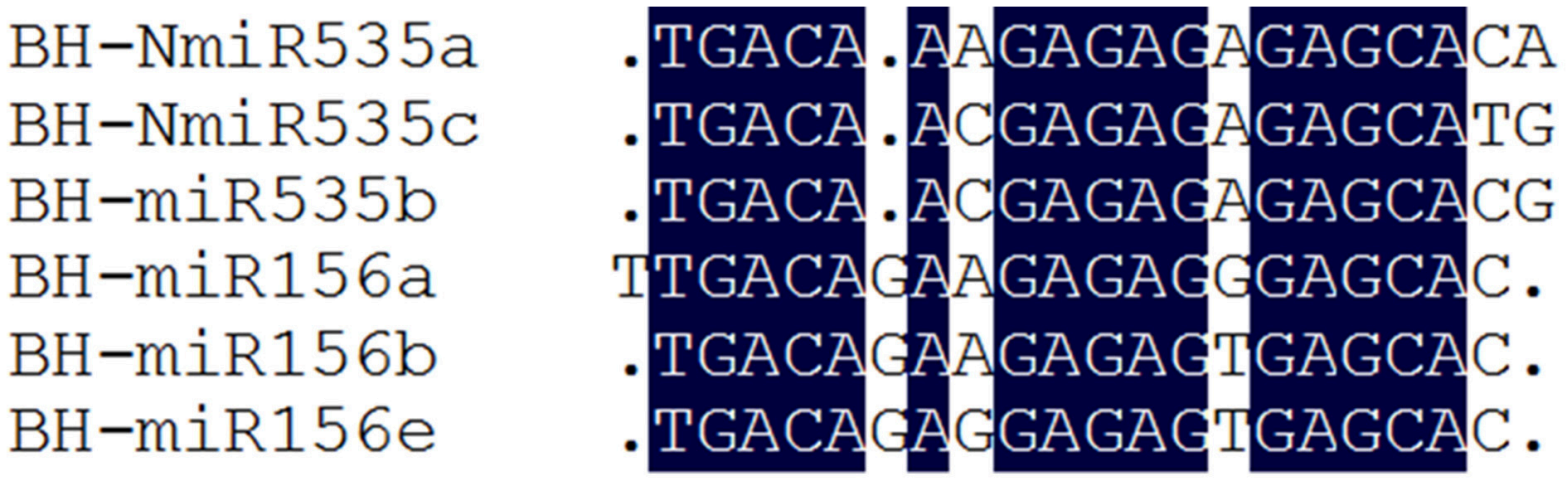
**Sequence alignment of miR535 and miR156 from “Baihe-35-1.”** The result revealed high similarity between vvi-miR156 and vvi-miR535 family in *Viti*, indicating possible evolutional and functional relationships. Mature miRNAs are shown in 5′ to 3′ orientation. Dark blue shading indicates nucleotides were conserved in 100% of the sequences.

To elucidate the function of miRNAs, it is essential to identify and characterize their targets. We employed the tool psRNATarget (http://plantgrn.noble.org/psRNATarget/) to identify the potential targets of all Chinese wild *V. pseudoreticulata* miRNAs. Finally a total of 485 experimental verified or putative transcripts were predicted for 214 vvi-miRNAs. Nine known miRNAs (vvi-miR399a, vvi-miR399h, vvi-miR396a, vvi-NewmiR403a, vvi-miR2111, vvi-miR2950, vvi-NewmiR4376, vvi-NewmiR399, vvi-NewmiR403b) and 24 candidates had no target mRNA predicted. Consistent with previous reports, many targets of conserved miRNA were transcription factors. For example, vvi-miR160 mainly targeted *ARF*s, vvi-miR171 targeted several members of the *SCL* (scarecrow-like) family. As reported by Carra et al. (2009), in our research, the mature sequence alignment (Figure 3) showed high similarity between vvi-miR535 and vvi-miR156 family from Chinese wild grape. This explains their same target prediction—the transcription factor *SPL* (squamosa promoter-binding-like protein). In contrast to conserved miRNAs, targets of non-conserved vvi-miRNAs had wider diversity. This indicates that it”s more likely that non-conserved miRNAs are functionally species specific.

**Figure 4:**
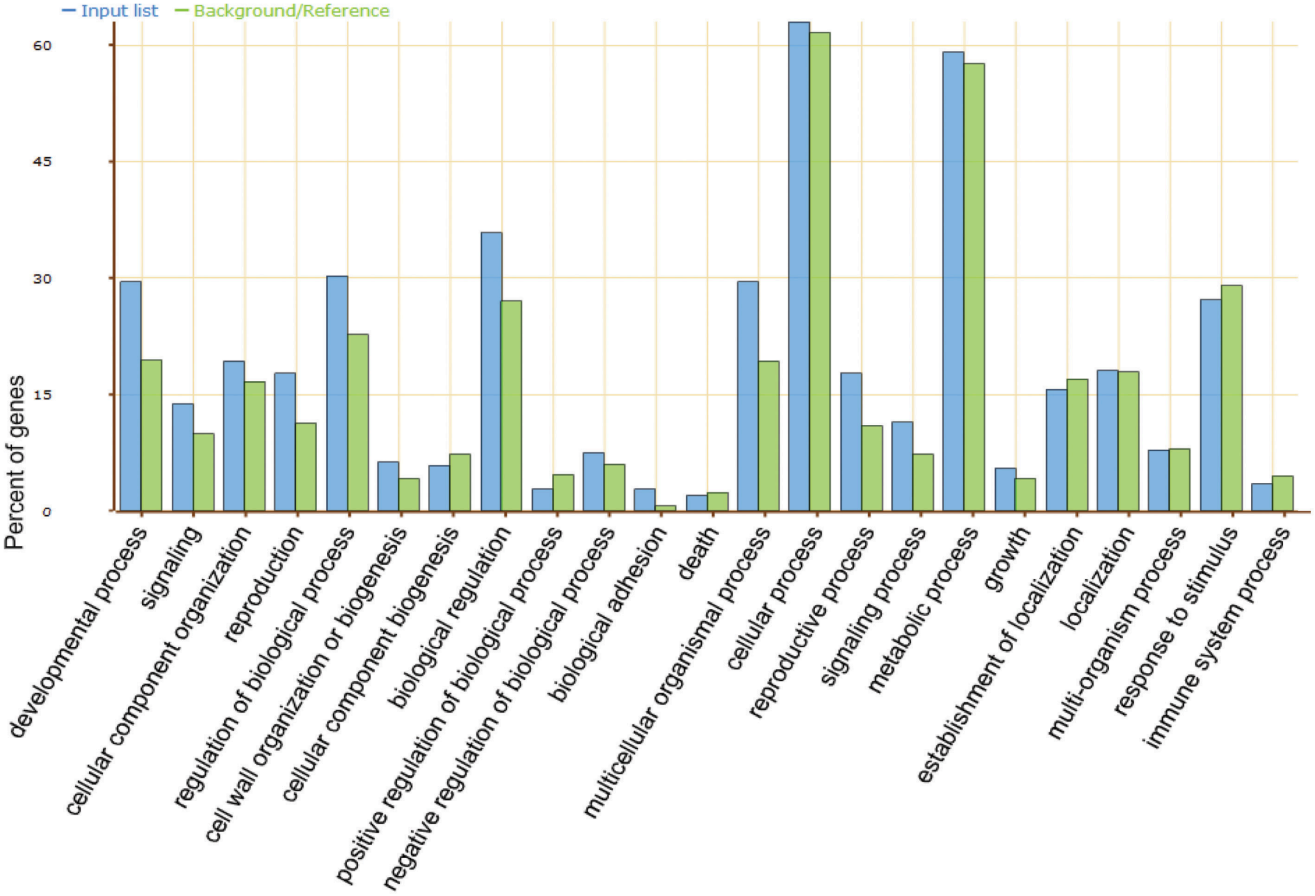
**GO annotation for all the predicted targets of miRNAs got from “Baihe-35-1**.”

Gene Ontology (GO) analysis (Figure 4) was carried out for all putative targets. According to the GO term identification, the molecular function involved mainly in binding, catalytic, transcription regulation, transporter, and molecular transducer activity. For the biological process, the putative target transcripts of miRNAs were classified into 23 categories, and involved mainly in growth and development, multicellular organismal process, metabolism and stimulus response. Based on GO terms and previous studies on R genes and genes related to defense responses, 49 disease resistance genes being targeted by 32 vvi-miRNA families (15 known and 17 candidates) were identified in our research. Remarkably, several miRNA families, including vvi-miR156, 159, 171, 172, 390, and 396, traditionally regulating the growth and development of plants were found with R or R-related gene targets.

### Expression and identification of miRNAs involved in powdery mildew resistance

When plants suffer environmental stresses, the expression of the miRNA repertoire often shows fluctuation. Here, the expression of miRNAs was standardized by RPKM (reads per kilo bases per million reads). We found 131 miRNAs were up-regulated and 119 were down-regulated after inoculation with *E. necator*. In our study, 32 vvi-miRNAs corresponding to 68 non-redundant sequences, displayed significant differential expressions (fold change ≥ 2 and *P* < 0.5), including 18 up-regulated and 11 down-regulated known miRNAs and 3 up-regulated candidates (vvi-New27, vvi-New 69a and vvi-New82; Table S5). Meanwhile, vvi-miR3636, vvi-miR3623, and vvi-New27 were only found in the experiment group, so their expression may require induction by pathogens. In addition, the expression of vvi-miR396b showed the largest rise, followed by vvi-miR164d and vvi-miR172d. The expression level of vvi-miR479 declined most, to 9% of normal level after inoculation with *E. necator*. Other significantly reduced miRNAs were vvi-MIR3633a, vvi-NewmiR535a, and vvi-NewmiR827. Vvi-MIR156a, 166a, 167c, vvi-NewmiR403a,b failed to be detected in the treated library.

**Figure 5:**
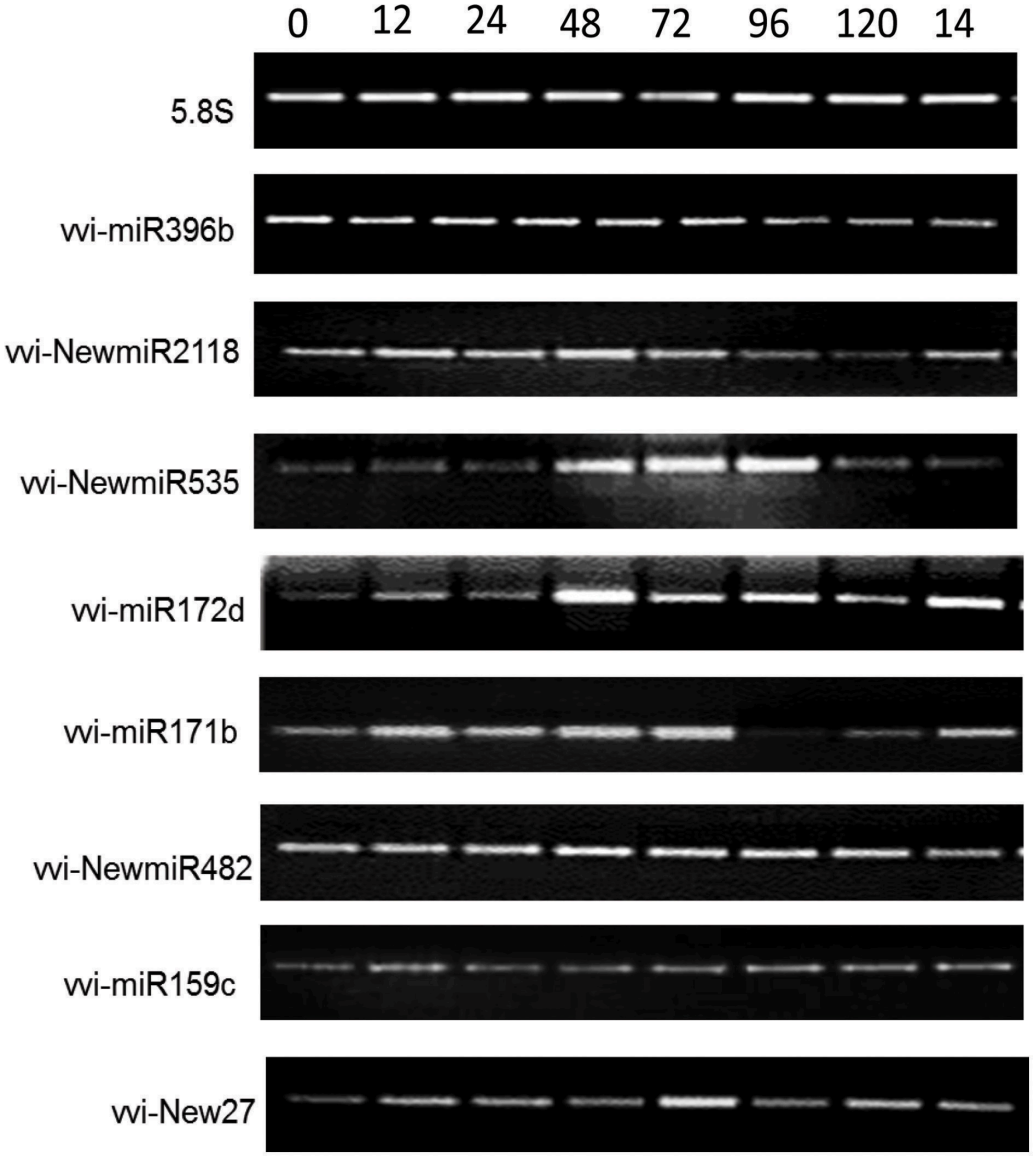
**Expression analysis by semi-quantitative RT-PCR of miRNA from “Baihe-35-1” after inoculation by ***Erysiphe necator*** for 0, 12, 24, 48, 72, 96, 120, and 144 h**. 5.8S rRNA was chosen as reference gene.

To verify our identification of miRNAs from wild Chinese *V. pseudoreticulata* and uncover their expression conditions, we performed semi-quantitative RT-PCR for candidate vvi-New27, conserved vvi-miR159c, 396b, 172d, 171b and non-conserved vvi-NewmiR535, NewmiR482, and NewmiR2118 (Figure 5). Before *E. necator* inoculation, vvi-miR396b, vvi-NewmiR2118 and vvi-NewmiR482 expressed strongly in leaves of “Baihe-35-1,” vvi-miR171b and vvi-New27 had obvious expression while vvi-miR159c, vvi-NewmiR535 and miR172d expressed weakly. After *E. necator* inoculation, the expressions of vvi-miR396b, vvi-NewmiR482, vvi-NewmiR535 and vvi-New27 did not change markedly, while vvi-NewmiR2118 and vvi-miR171b reduced sharply after 96 h, vvi-miR172d was down-regulated after 48 h, the expression of vvi-NewmiR535 was up-regulated soon after inoculation for 48 h and such high expression lasted for 3 days before reducing rapidly to its normal level by about 120 h after inoculation. The expression patterns of vvi-miR172d, vvi-NewmiR2118, vvi-NewmiR482, vvi-miR159c, vvi-New27, and vvi-NewmiR535 analyzed by semi-quantitative RT-PCR and miRDeep-P were consistent but for vvi-miR396b and vvi-miR171b.

**Figure 6:**
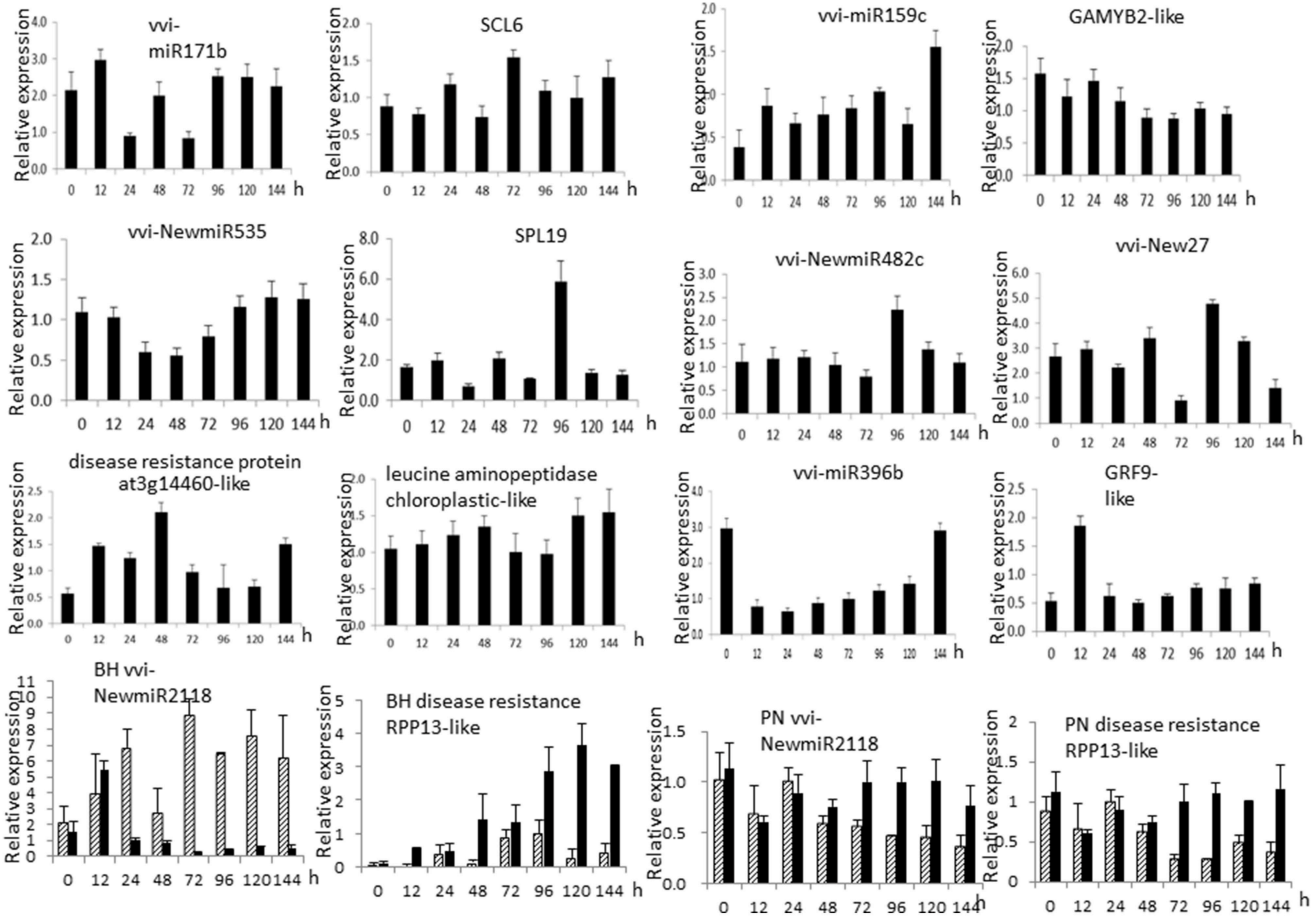
**qRT-PCR of miRNA (left) and targets (right)**. 0, 12, 24, 48, 72, 96, 120, and 144 h mean the time when samples were collected after inoculation with *Erysiphe necator*. 5.8S rRNA was chosen as internal reference.

Apart from semi-quantitative RT-PCR, real-time qRT-PCR (qRT-PCR) was also done for several miRNAs and their targets (Figure 6). The expression of vvi-miR171b, vvi-NewmiR2118, vvi-NewmiR535, and vvi-New27 in Chinese wild grape was reduced noticeably after pathogen infection, with the main reduction occurring 24–72 h after inoculation. The accumulation of vvi-miR396 decreased markedly 12 h after inoculation but then gradually recovered to its normal level. Vvi-NewmiR2118 was fast and markedly down-regulated during after inoculated 24 h in Baihe-35-1, while in susceptible grapevine Piont Noir its expression did not show obvious changes. So we suggested the involvement of vvi-NewmiR2118 from *V. pseudoreticulata* in resistance to powdery mildew. The expressions of vvi-NewmiR482 and vvi-miR159c fluctuated gently after pathogen infection. Except vvi-miR396b, vvi-NewmiR535, and vvi-New27, the expression patterns of most miRNAs examined by semi-quantitative RT-PCR and qRT-PCR were similar.

Since plant miRNA negatively regulates its target(s), qRT-PCR was also carried out for some target genes (Figure 6). Results displayed a negative relationship between vvi-miR171b and target *SCL*6 (GSVIVT0102768000, scarecrow-like protein 6), which also applied to vvi-miR159c and its target *GAMYB-like* (GSVIVT01012447001); vvi-miR396b and its two targets-GRF9 (GSVIVT01027535001, growth-regulating factor 9-like) and leucine aminopeptidase chloroplastic-like (GSVIVT01023604001) expressed stably except at some individual test points. The expression of vvi-NewmiR535 and target *SPL9* (GSVIVT01033519001, squamosa promoter binding protein 9-like) did not show clear relation. However, there was obvious opposite trend in the expression changes of vvi-NewmiR2118 and its two targets: disease resistance protein at3g14460-like (GSVIVT01001446001) and disease resistance protein RPP13-like (GSVIVT01013307001).

Based on the differential expression and GO term(s) of target gene(s), 13 known vvi-miRNA families and one candidate vvi-New69a with 36 targets were identified as miRNAs that may be involved in enhanced powdery mildew resistance in wild Chinese *V. pseudoreticulata* (Table 2). And their targets were either R genes or signal-related genes involved in biotic stress.

**Table 2:** **vvi-miRNAs involved in powdery mildew resistance of wild Chinese ***Vitis pseudoreticulata*****.

**vvi-miR**	**Target**	**Target desc**.
vvi-MIR156f	GSVIVT01016962001	Probable disease resistance protein at5g63020-like
vvi-MIR159c	GSVIVT01010961001	Zinc finger protein MAGPIE-like
	GSVIVT01037667001	r2r3-myb transcription
	GSVIVT01001155001	Copalyl diphosphate synthase
vvi-MIR164d	GSVIVT01016290001	Para-aminobenzoate synthase-like
vvi-MIR166a	GSVIVT01034812001	TMV resistance protein n-like
vvi-MIR166f	GSVIVT01034812001	TMV resistance protein n-like
vvi-MIR172d	GSVIVT01016352001	Ethylene-responsive transcription factor rap2-7-like
	GSVIVT01025100001	Ethylene-responsive transcription factor rap2-7-like
vvi-MIR3633a	GSVIVT01023557001	Disease resistance protein at4g27190-like
vvi-MIR390	GSVIVT01015035001	Auxin response factor 19-like
	GSVIVT01038628001	Leucine rich repeat family expressed
vvi-MIR396b	GSVIVT01035367001	Receptor-like protein kinase hsl1-like
	GSVIVT01037219001	TMV resistance protein n-like
vvi-MIR479	GSVIVT01035459001	Transcription repressor myb4
vvi-New69a	GSVIVT01003955001	1-deoxy-d-xylμlose 5-phosphate synthase
vvi-NewmiR2118	GSVIVT01001446001	Disease resistance protein at3g14460-like
	GSVIVT01007026001	Disease resistance protein at3g14460-like
	GSVIVT01013307001	Disease resistance ΔRPP13-like protein 1-like
	GSVIVT01014595001	Disease resistance protein at4g27190-like
	GSVIVT01028627001	Disease resistance protein rpm1-like
	GSVIVT01029009001	TMV resistance protein n-like
vvi-NewmiR482	GSVIVT01033046001	Della protein gai1-like
vvi-NewmiR535a	GSVIVT01011939001	Mediator of rna polymerase ii transcription subunit 25-like

Each list from left to right represented miRNAs involved in powdery mildew resistance from “Baihe-35-1,” targets relative to defense or resistance responses and their description.

### Characteristics of vvi-NewmiR2118 and its targets

**Figure 7:**
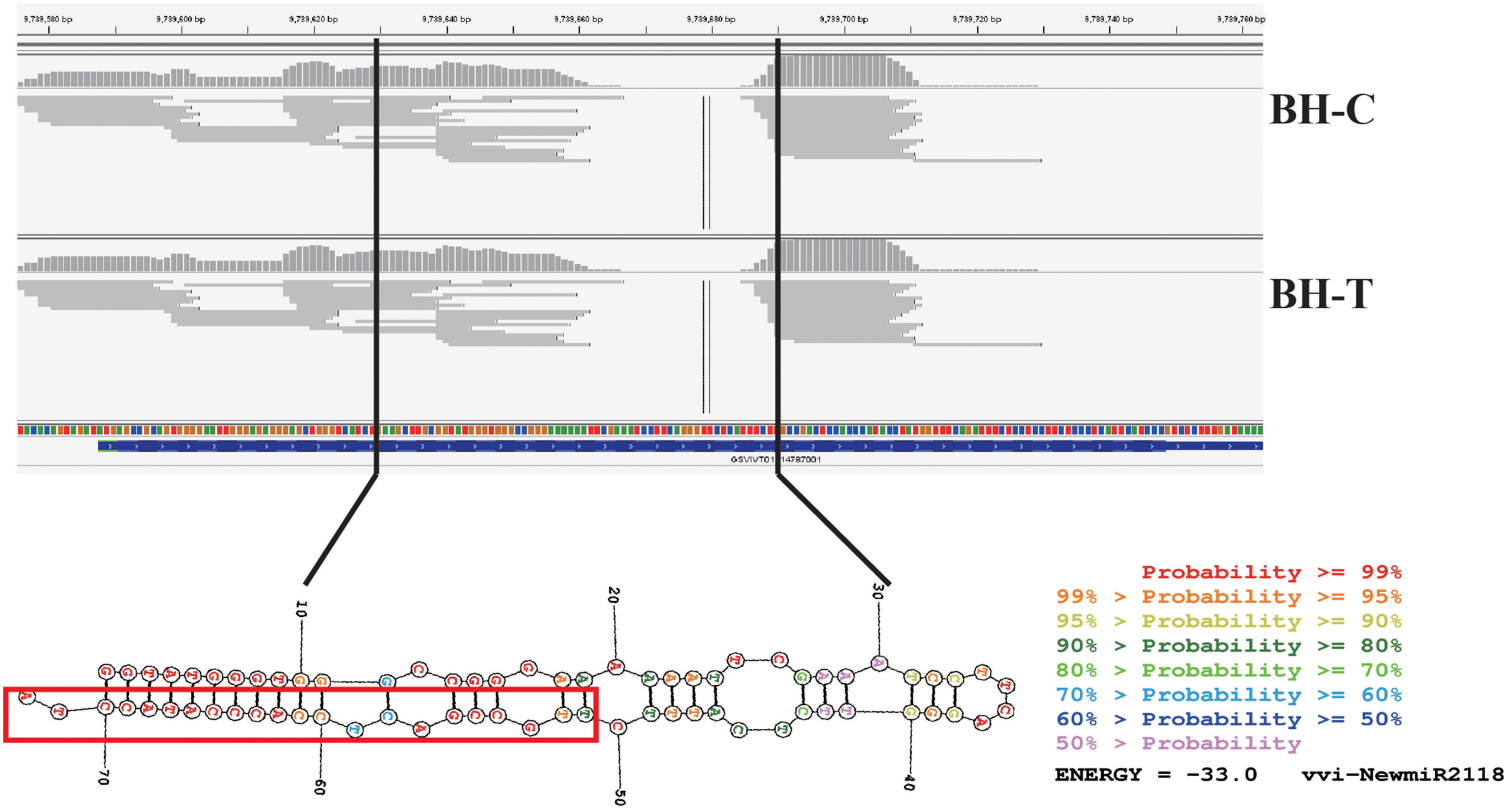
**Signature distribution of vvi-NewmiRNA2118 reads on Chinese wild grape genome and hairpin-like secondary structure of its precursor sequence**. The color indicates base pair probabilities. The part in the box shows the mature sequence of vvi-NewmiRNA2118.

Among the miRNAs from wild Chinese *V. pseudoreticulata* predicted to be involved in powdery mildew resistance, vvi-NewmiR2118 was most interesting. Its expression decreased quickly and strongly in infected leaves and its predicted targets were all NBS-LRR type R genes. In “Baihe-35-1,” the gene *MIR2118* was located on chromosome 19 and its precursor sequence was located from 9739640 to 9739711. The reads of vvi-NewmiR2118 showed significant accumulation on the gene *GSVIVT01014787001*, providing reliable information about the transcription and process of the precursor of miR2118 (Figure 7).

**Figure 8:**
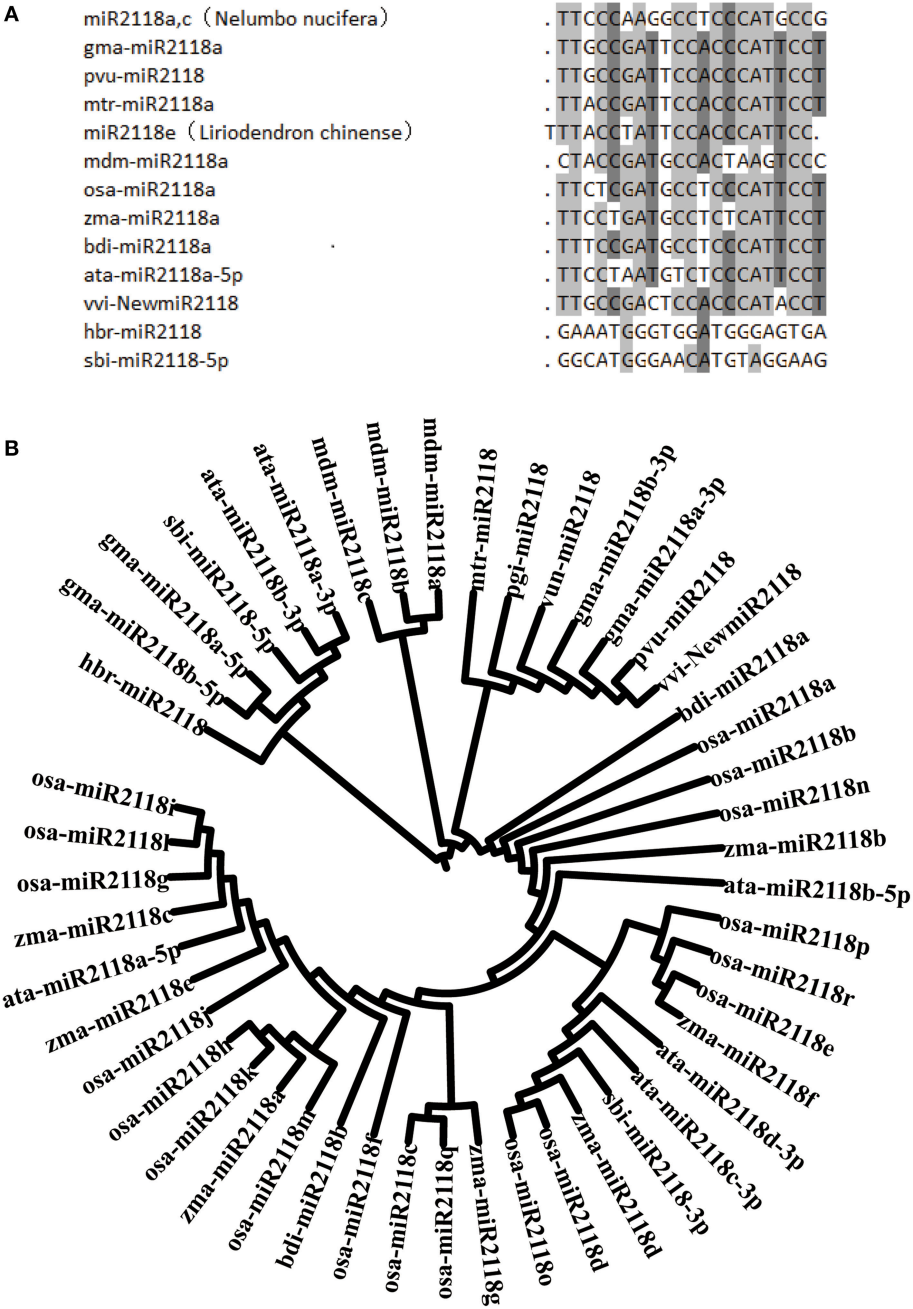
**Sequence alignment (A) and phylogeny analysis (B) for vvi-NewmiR2118. (A)** 13 sequences for alignment were from the following species: *Nelumbo nucifera* (Zheng et al., 2013), *Phaseolus vulgaris* (Arenas-Huertero et al., 2009), *Glycine max* (Kulcheski et al., 2011), *Medicago truncatula* (Jagadeeswaran et al., 2009b), *Zea mays* (Johnson et al., 2009), *Oryza sativa L.* (Johnson et al., 2009), *Brachypodium distachyon* (Jeong et al., 2013), *Aegilops tauschii* (Jia et al., 2013), *Malus domestica* (Xia et al., 2012), *Hevea brasiliensis L.* (Lertpanyasampatha et al., 2012), *sugarcane* (Thiebaut et al., 2012), *Liriodendron chinense* (Wang et al., 2012), and Chinese wild *Vitis pseudoreticulata*. Mature miRNAs were shown from 5′ to 3′ end. Light gray and dark gray shading indicates the nucleotides were conserved in more than 70% or more than 50% (< 70%) of the sequences, respectively. **(B)** There were 47 miRNA2118 sequences from miRBase 21.0 used for phylogeny analysis. As shown in the figure, annual and perennial plants clustered separately. vvi-NewmiR2118 was most closely related with that of *Malus domestica* and *Panax ginseng*, and have close relations with *Phaseolus vulgaris*.

The conservative characteristics of miR2118 were also analyzed by blasting sequences of miR2118 from 13 species (Figure 8A). The alignment result indicated that miR2118 is pretty conserved in plants except frequent base changes at the third and ninth position from the 5′ end. The bases at the eleventh and sixteenth position (according to vvi-NewmiR2118) were almost fixed with C and A, respectively. Multiple alignment of vvi-NewmiR2118 and all 47 published miR2118 sequences from miRBase 21.0 (Figure 8B) showed that miR2118 from annual plants such as *Zea mays, Oryza sativa* L. and *Aegilops tauschii* were closely related, while miRNAs from perennial plants were clustered. Particularly, vvi-NewmiR2118 shared sequence identity with *Vigna unguiculata, Phaseolus vulgaris*, and *Panax ginseng*.

**Figure 9:**
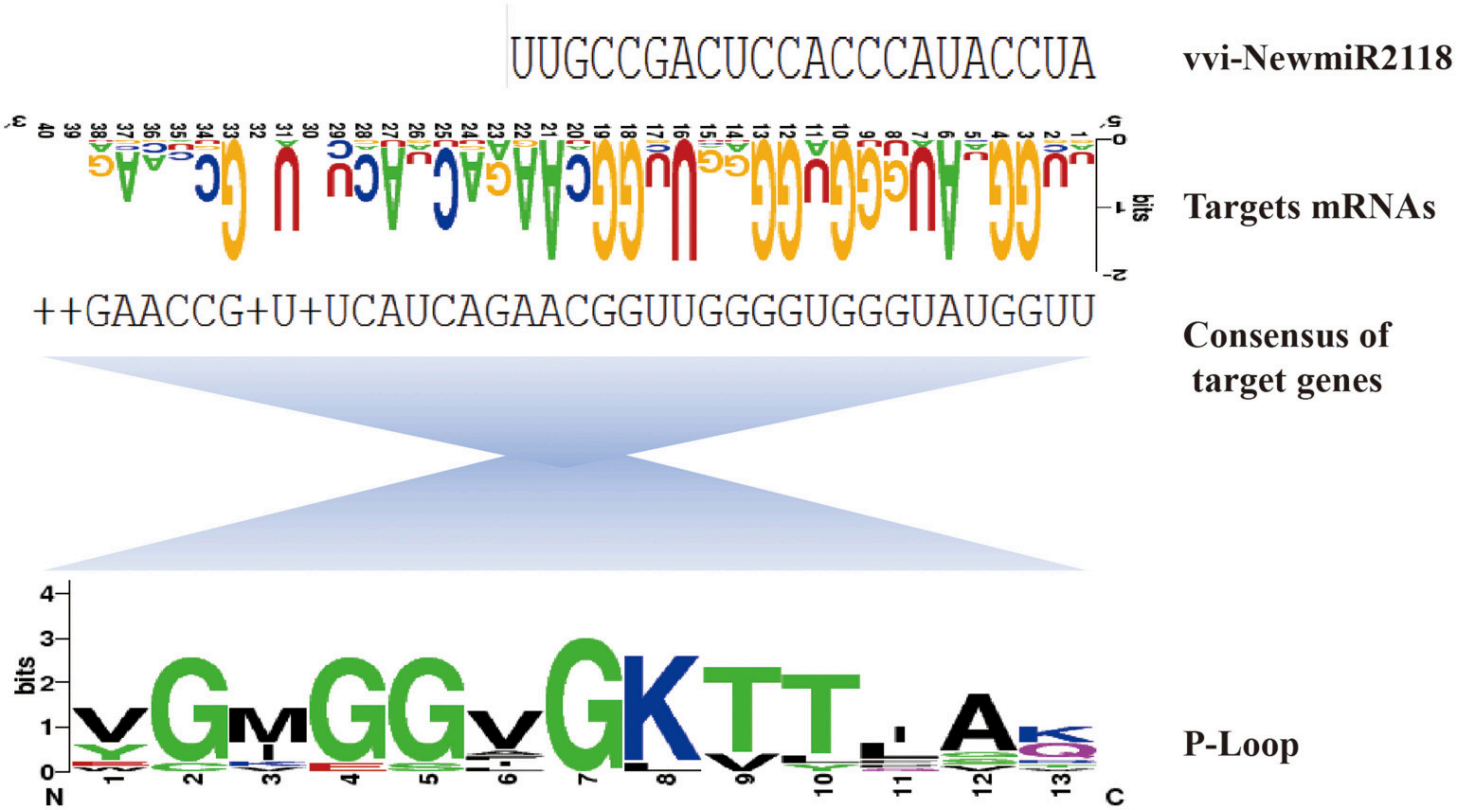
**Analysis of the interaction region of vvi-NewmiR2118 with NB-LRR targets**. vvi-NewmiR2118 (first line, shown in 5′ to 3′ orientation) bound on the conserved p-loop motif (third line, translates of target genes) of NB-LRR targets (second line).

We predicted eight targets for vvi-NewmiR2118. Among them, five were NBS-LRR disease resistance genes containing NB-ARC and LRR domains (GSVIVT01001446001putative disease resistance protein At3g14460-like; GSVIVT01007026001 putative disease resistance protein At3g14460-like; GSVIVT01014595001 disease resistance protein At4g27190-like; GSVIVT01028627001 disease resistance protein RPM1-like; GSVIVT01013307001 disease resistance RPP13-like protein1) and one was the TMV resistance protein N-like (GSVIVT01029009001). The 3′ end of vvi-NewmiR2118 bound with the conserved p-loop motif of all targets, except the disease resistance protein RPM1-like (Figure 9).

### Verification of the interaction between vvi-NewmiR2118 and RPP13

Disease resistance gene *RPP13* (Recognition of *Peronospora parasitica*, RPP) was one of the predicted targets of vvi-NewmiR2118, whose expression could be inhibited by degrading the mRNA. We transiently co-expressed vvi-NewmiR2118 and ΔRPP13-GUS in tobacco leaves and verified their interaction by histochemical staining and GUS fluorescence quantitative assay.

**Figure 10:**
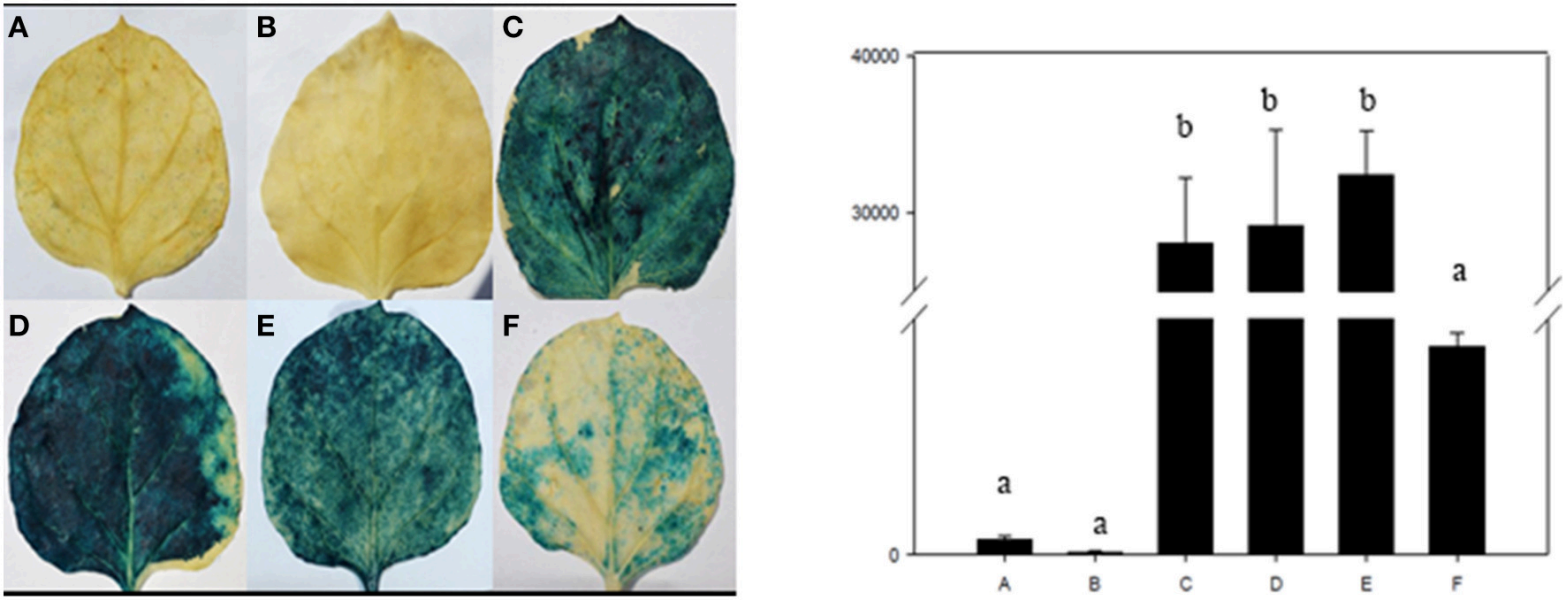
**GUS accumulation by histochemical staining (left) and fluorescence quantitative assay (right) of transiently transformed tobacco leaves mediated by ***Agrobacterium*** : (A)** penetration buffer, **(B)** Pro35S:MIR2118, **(C)** Pro35S:GUS, **(D)** Pro35S:ΔRPP13-GUS, **(E)** Pro35S:MIR2118 and Pro35S:GUS (v/v = 5:1), and **(F)** Pro35S:MIR2118 and Pro35S:ΔRPP13-GUS (v/v = 5:1).

Based on histochemical staining (Figure 10), *GUS* expressed strongly when transformed alone into tobacco leaves (C,D). This also happened in leaves co-transformed by *Pro*_35*S*_*:MIR2118* and Pro_35*S*_*:GUS* (v/v = 5:1) (E) because vvi-NewmiR2118 could not silence GUS due to the deficiency of interaction site. But in leaves co-transformed by *Pro*_35*S*_*:MIR2118* and *Pro*_35*S*_*:*Δ*RPP13-GUS* (v/v = 5:1) (F), only very weak GUS expression was found. The explanation was that Δ*RPP13* introduced target sequence which could be identified and cleaved by vvi-NewmiR2118, which as a result blocked the expression of *GUS*. So GUS staining verified the negative regulation of vvi-NewmiR2118 to ΔRPP13.

The differences in GUS protein activity in each treatment were further analyzed by fluorescence quantitative assay. There were no significant difference in GUS activity among the control and the combination of *Pro*_35*S*_*:MIR2118* and *Pro*_35*S*_*:*Δ*RPP13-GUS*. This also occurred with leaves transformed by *Pro*_35*S*_*:GUS, Pro*_35*S*_*:*Δ*RPP13-GUS* and leaves co-transformed by *Pro*_35*S*_*:MIR2118* and *Pro*_35*S*_*:GUS*. However, the GUS activity was significantly different between the two groups. So the GUS fluorescence quantitative assay supported the results of histochemical staining. Based on these series of experiments, we believed that RPP13 was a real target of vvi-NewmiR2118.

## Discussion

Small RNA high-throughput sequencing is now a widely-used method for miRNA screening, which has resulted in the discovery of a huge number of sRNA from a wide range of plant species. Therefore in this study, we also adopted this method to identify miRNAs in Chinese wild *V. pseudoreticulata*. The common lengths of plant sRNA are usually 21 or 24 nt (Rajagopalan et al., 2006; Fahlgren et al., 2007; Moxon et al., 2008). In many plants, the number of 24nt sRNA usually exceed 21nt sRNA, for example, in gymnosperms *Taxus chinensis* (Qiu et al., 2009), in monocots *Oryza sativa* (Morin et al., 2008) and in eudicots *Arabidopsis* (Rajagopalan et al., 2006), tomato (Moxon et al., 2008), and *Citrus trifoliata* (Song et al., 2010). But according to our results, the 21nt sRNAs were more abundant than the 24nt ones in the leaf tissue of wild Chinese *V. pseudoreticulata*, which contrasts with many other plants. In other grapevine species *Populus balsamifer* (Barakat et al., 2007), *Pinus cordata* (Morin et al., 2008) and in leaves, berries, tendrils and flowers of Pinot Noir (Pantaleo et al., 2010), the 21nt class of sRNAs also showed higher abundance. Since 21 and 24nt are the typical lengths of miRNA and siRNA, respectively, we suggest the type of sRNA playing dominant roles in plant species may be different, with annuals more generally having 24nt miRNA and siRNA, and perennials 21nt miRNA.

MiRDeep-P is an effective and efficient tool for plant miRNA identification. In this research, miRDeep-P combined with sRNA high-throughput sequencing not only detected known vvi-miRNAs, but also predicted 124 candidate miRNAs from leaves of “Baihe-35-1” under *E. necator* inoculation condition. Previous studies have reported several novel miRNAs from the *V. vinifera* grapevine cultivars Corvina and Pinot Noir respectively (Mica et al., 2009; Pantaleo et al., 2010). Meanwhile, another study describes new miRNAs from the interspecific table grape cultivar Summer Black (Wang et al., 2011), thus enlarging the vvi-miRNA repertoire. However, not all these reported novel miRNAs were found from “Baihe-35-1.” This may be due to the tissue and stress condition we used to construct the libraries. The different genetic backgrounds of the wild Chinese *V. pseudoreticulata*, Pinot Noir and Corvina could also explain this phenomenon. The 3′ ends of mature sequences in some miRNA families, including vvi-miR167b/c/d, vvi-miR171b/e and vvi-miR396a/b/c, contain one to three bases variation. We think this is possibly caused by inaccurate cleavage of DCL1 to miRNA precursors.

An interesting finding in our research is the high sequence similarity between vvi-miR156 and vvi-NewmiR535. Since the conserved miRNA evolved earlier than the non-conserved, we suggest that vvi-miR156 may be the ancestor of vvi-NewmiR535 and form a superfamily. However, many studies prove that the SPL family is mainly regulated by miR156 (Xie et al., 2006), indicating that non-conserved miRNAs have evolved new and different functions from their ancestors.

In this research, a large number of target genes have been predicted for miRNAs. In terms of conserved miRNAs, their targets were mainly transcription factors, which agrees with previous studies. The origin of plant miRNA dates back 400 million years (Floyd and Bowman, 2004) and their targets primarily regulate the growth and development of plants, indicating the key role of miRNA in plant survival. However, conserved miRNAs do not equal that their functions are also conserved. For example, each of the three conserved miRNA families, miR473a, miR478a, and miR482 from *Populus* and rice has different functions (Lu et al., 2005). In this study, we predicted targets which are not transcription factors for some conserved vvi-miRANs as well. For example, vvi-miR172c had a disease resistance target *RPM-like* (GSVIVT01028632001); vvi-miR156 and vvi-miR396 targeted disease resistance related protein GSVIVT01016962001 and TMV resistance protein n-like (GSVIVT01037219001). We suggest these conserved miRNA may be involved in stress responses. In contrast with conserved miRNAs, the targets of non-conserved miRNAs varied greatly, explaining their diverse roles in biological processes.

By semi-quantitative RT-PCR and qRT-PCR, we uncovered the expression patterns of a part of those identified miRNAs from Chinese wild *V. pseudoreticulata* after *E. necator* inoculation. Results showed that many miRNAs (vvi-miR159c, vvi-miR164, vvi-miR166f/g, vvi-miR396b, vvi-miR171, vvi-miR172, vvi-miR156, vvi-miR166a, vvi-miR390) believed previously mainly involved in growth and development could be affected when suffered from fungi stress. Sunkar et al. (2012) also show that stress can significantly alter the expression profiles of most miRNAs function in growth and development. Our prediction of R or R-related target genes for vvi-miR156, 159, 171, 172, 390, and 396 may partially help explain their fluctuating expression. On the other hand, this may also provide information about why the normal growth status can be altered under biotic stress. For example, miR160, miR167 with targets of *ARFs* and miR393 with target of *TIR1* are all up-regulated, while miR390 is down regulated under a range of stress conditions. Nevertheless, their common effect is the suppression of auxin signaling and growth (Sunkar et al., 2012).

In terms of the expression pattern of vvi-miRNAs, our results were not consistent when analyzed by miRDeep-P, semi-quantitive RT-PCR, and qRT-PCR. The most likely reason comes from samples used for sRNA high-throughput sequencing. When we built the “Baihe-35-1” library under *E. necator* stress, we mixed equal total RNA extracted from all individual leave samples inoculated with *E. necator* for different time. It may be that differential expression of some vvi-miRNAs has been balanced by opposite-going changes at different time points.

The R genes are very important in plant genome. NBS-LRR genes form overwhelming majority of them. NBS-LRR proteins are typically intracellular, soluble proteins with three domains: an N-terminal signaling domain, a central nucleotide binding site domain, and C-terminal leucine-rich repeats (LRR) (Jones and Dangl, 2006). Nucleotide binding site (NBS) contains three domains: p-loop (kinase-a), kinase-2a, and kinase-3a. The signature sequence of the p-loop domain is GM (G/P)GXGKTT(a/r). According to Velasco et al. (2007), several clusters of NBS genes mapped to chromosomal regions were previously assigned to genetic resistance to fungal diseases such as downy and powdery mildews in grape. Many targets of miR2118 are often NBS-LRR genes, implying that miR2118 plays a role in host-pathogen interactions (Jagadeeswaran et al., 2009b; Zhai et al., 2011; Shivaprasad et al., 2012). Research on tobacco and *Glycine max* shows that miR2118 usually targets the conserved regions, mainly p-loop sequences in these genes and functions by the generation of trans-acting siRNAs (Zhai et al., 2011).

To date, the function of miR2118 in grape and other plants is still poorly understood and its target mRNAs have not been identified. In our research, we first identified 22nt long vvi-NewmiR2118 in wild Chinese *V. pseudoreticulata* and its expression was very high. When the wild grape suffers fungi attack, vvi-NewmiR2118 will be quickly downregulated. Moreover, we predicted targets for vvi-NewmiR2118. In accordance with many previous reports, these target genes were all R genes of the NBS-LRR type. Experiments also proved interaction of vvi-NewmiR2118 and resistance gene *RPP13*. However, it's still unknown how this miRNA functions in this pathway and if it will mediate the biosynthesis siRNAs. We analyzed the binding region of vvi-NewmiR2118 with several of its NBS-LRR targets and found that in grape, miR2118 also bound onto the p-loop domain of its targets. Hence we speculate that vvi-NewmiR2118 may regulate its target gene by producing pha-siRNA, however, more work is required to verify this idea.

## Conclusion

To explore the defense mechanisms of grape to powdery mildew at the level of post-transcription regulation, we identified miRNAs from highly resistant wild grapevine “Baihe-35-1.” Combined with sRNA high-throughput sequencing, bioinformatics and molecular biology technologies, we profiled and identified vvi-miRNAs that might be involved in powdery mildew resistance in Chinese wild *V. pseudoreticulata*. This not only revealed a novel layer of defense mechanisms against biotic stresses employed by grape, but also provided a new method for breeding disease resistant grapevines through transgenic technology.

## Author contributions

YX conceived and initiated the work; LH designed the experiments, LH, HM carried out the experiments. LH analyzed the data and wrote the paper; KW, GX, and GL carried out and supervised the computational work. LH, KW are co-authors.

### Conflict of interest statement

The authors declare that the research was conducted in the absence of any commercial or financial relationships that could be construed as a potential conflict of interest.

## Supplementary material

Table S1 **Primers used in this study**.

Table S2 **vvi-miRNAs identified in the leaf of “Baihe-35-1**.”

Table S3 **vvi-miRNAs detected only in one library**.

Table S4 **List of conserved miRNAs obtained from leaves of “Baihe-35-1**.”

Table S5 **Differentially expressed miRNAs from leaves of “Baihe-35-1” after inoculation with ***Erysiphe necator*****.
